# Mid-career pitfall of consecutive success in science

**DOI:** 10.1038/s41598-024-77206-y

**Published:** 2024-11-15

**Authors:** Noriyuki Higashide, Takahiro Miura, Yuta Tomokiyo, Kimitaka Asatani, Ichiro Sakata

**Affiliations:** Department of Technology Management for Innovation, Graduate School of Engineering, The University of Tokyo, Tokyo, 113-8656 Japan

**Keywords:** Statistical physics, thermodynamics and nonlinear dynamics, Computational science, Scientific data, Statistics

## Abstract

The creativity of scientists often manifests as localized hot streaks of significant success. Understanding the underlying mechanisms of these influential phases can enhance the effectiveness of support systems and funding allocation, fostering groundbreaking discoveries worthy of accolades. Historically, analyses have suggested that hot streaks occur randomly over time. However, our research, through meticulous examination, reveals that these phases are not flatly distributed but are more frequent at the early and late stages of scientists’ careers. Notably, both early and late hot streaks are marked by dense tie collaborations, with the former typically involving close partnerships with particular authors and the latter being characterized by involvement in large-scale projects compared with single-top or ordinary papers. This pattern indicates that mid-career researchers lack both intimate relations and resources to keep big projects, leading to “mid-career pitfall” of consecutive success. This insight holds profound implications for the development of policies and initiatives aimed at bolstering innovative research and discovery.

## Introduction

Over the past half-century, the role of scientists has expanded beyond simply writing high-quality papers, making it more difficult to focus solely on academic interests due to the growing need for broader engagement in academia. University faculty members are increasingly busy with committee meetings for selecting other faculty members and Ph.D. candidates, grant reports and presentations, teaching, meetings with lab team members, trips to discuss with external collaborators, and attending a kick-off event for a new research consortium^1^. This diversification of a scientist’s skill set dilutes the resources available for accelerating sciences^2^, even emerging the possibility that the labor advantage in scientific fields could be becoming a bottleneck in driving productivity^3^. Given the escalating challenges in achieving remarkable results with limited time and resources, it is important to understand the mechanisms that enable scientists to be actively engaged in their work from systematic and ecosystem-based perspective. This understanding is crucial for research support, including those involved in research management initiatives, funding, and science and technology policy, as it poses a significant challenge that needs to be clearly addressed.

The phenomenon known as hot streaks^4,5^ is one of the distinguishing markers of a researcher’s engagement. Einstein’s “miracle year,” where he made four revolutionary discoveries, are typical example of a hot streak. New ideas often do not arise in isolation but are part of a chain where one discovery sparks another. Previous research shows that scientists’ top three papers are typically produced in a consecutive sequence^4^, so scientific success somewhat manifests with a curious continuity. Triggers for consecutive success may be more valuable than triggers for a singular success because single success may merely represent the fortunate extraction of a good idea from a universal distribution of ideas^6^, whereas the hot streak can be seen as a ‘bonus time’ that elevates the impact of every output over a certain period^4^. Thus, uncovering the universal patterns of hot streaks can help efficiently boost researchers’ intellectual creativity, potentially revolutionizing how we stimulate and sustain scientific innovation.

In investigating the mechanisms that could trigger hot streaks, a type of career success, the career ages should be considered^7^. Hot streaks are supposed to occur with a certain probability throughout any career ages^4^, but if we assume this legitimacy, consistently publishing consecutive high-impact works during the mid-career stage seems quite challenging due to the large number of involved projects, heavy workloads, funding issues, and other factors that demand attention. These challenges are explained in Nature Career’s special podcast series *“Muddle of the Middle”*^8^. In their mid-career, having recently earned their Ph.D. and within the first few years of obtaining tenure, face a unique set of challenges compared to their younger counterparts who often enjoy more funding and support, or even harder than senior scientists who have settled assets and experienced skills to manage various activities. These mid-career researchers are required to juggle an array of responsibilities, including managing their first teams with limited budgets, preparing new lectures, and consistently publishing papers to secure their next positions. Consequently, mid-career researchers may find it difficult to allocate enough time for both exploration and exploitation which often marks the onsets of hot streaks^5^.

It’s valuable to examine how hot streaks, *i.e.* consecutive successes, of scientists differ by career stages. Scientists’ academic career successes are related to their collaboration patterns such as team formations and co-authoring^9–19^. Hot streaks are more likely to be produced by larger teams^5^, but team composition approaches vary among career stages. Early-career researchers might be likely to focus on building fresh connections while working closely with their supervisors^12–14^. In contrast, senior researchers might leverage their established networks with previous collaborators and effectively manage super-ties, ensuring long-term collaboration^9,18^. What kind of collaboration produces consecutive success?

In this study, we investigated when top papers are produced in a researcher’s career and what factors are behind their creation depending on career stages, using a large-scale literature database. Contrary to previous studies, our results revealed U-shaped patterns, indicating that hot streaks are more common in the early and later stages of a career. For the early and later stages, we clarified differences in supporting factors of team collaboration. This allows for a better understanding of the mechanisms that keep scientists actively engaged in their work from a systematic and ecosystem-based perspective which can be useful insights for research policymakers, funding institutions, academic leaders.

## Consecutive successes concentrate on a career’s early and late stages

**Fig. 1:**
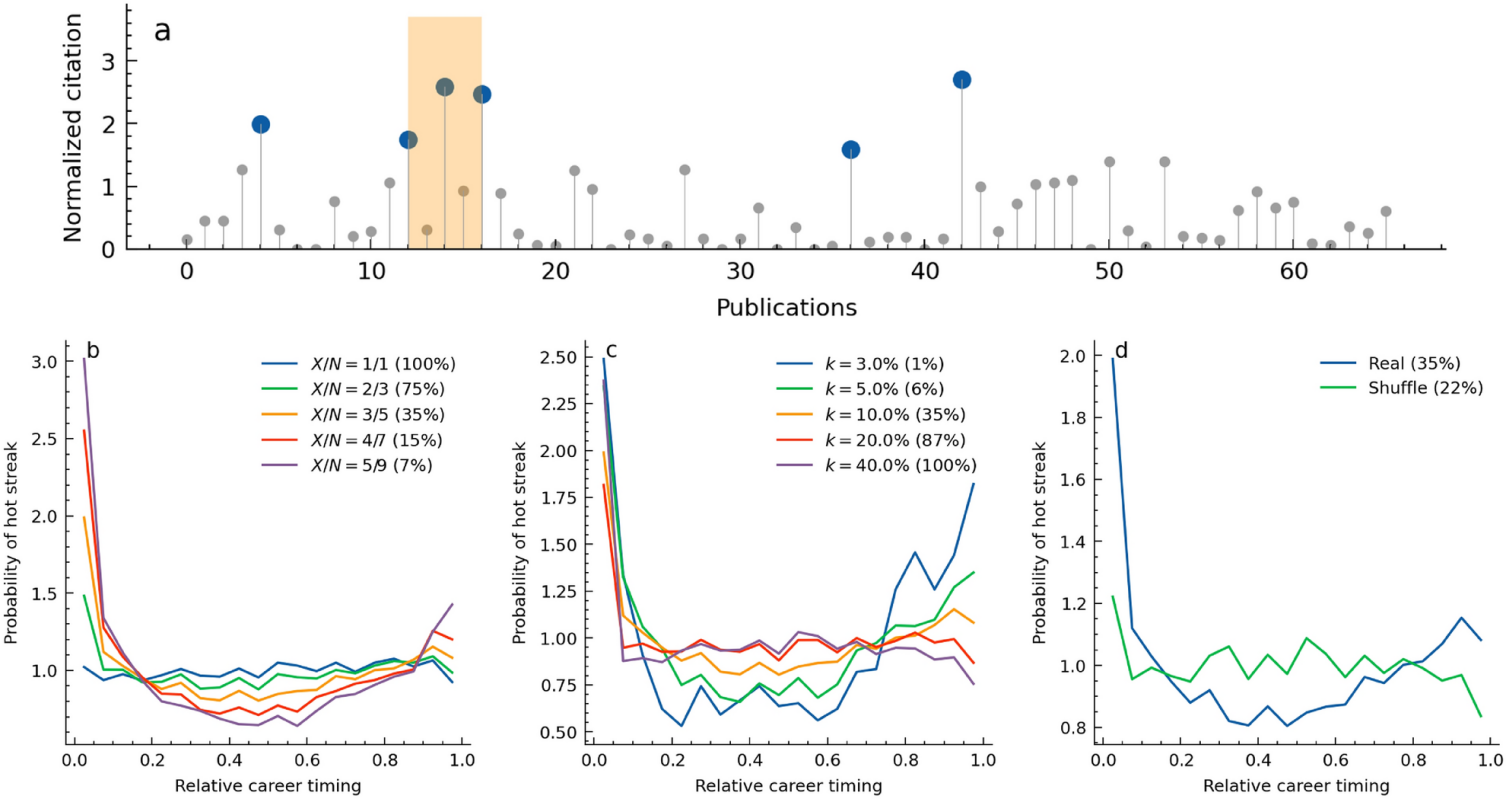
(**a**) Definition of consecutive success. Top *k*% impactful papers are shown as blue circles and they appear *X* times within *N* publications with parameters $X=3, N=5, k=10\%$. We use the number of publications in sequence as the length of the researchers’ career instead of years. (**b**-**d**) Probability distribution when consecutive success occurs in the scientists’ careers. The x-axis, relative timing, represents normalized career length. The numbers in parentheses represent the percentages of researchers, out of 100,000, who have experienced at least one hot streak under that parameter. As consecutive success becomes stronger, the number of researchers experiencing consecutive success decreases. (**b**) The distributions of consecutive success with different length-related parameters *X* and *N* with $k=10\%$. A single success ($X/N=1/1$) occurs with a constant probability but long continuous successes ($X/N=5/9$) occur more frequently in the early and late throughout the career. (**c**) The distributions of consecutive success for different *k* with $X/N=3/5$. Big success occurs more in the early and late stages. (**d**) compares the distribution between the raw and shuffled career sequences with parameters $X/N=3/5$ and $k=10\%$.

A researcher’s career history of publication impact is used to identify consecutive success. The career sequence dataset was crafted from over 100,000 scientists with careers spanning more than 20 years and 30 publications (Supplementary information 1.1). We defined a consecutive success as a duration when scientists’ top-*k*% most impactful papers are concentrated *X* times within *N* publications (see Methods for mathematical descriptions). In Figure 1a, for example, the top $10\%$ high-impact works indicated by blue circles, are concentrated with $X=3$ out of $N=5$ works in the period from 12 to 16 marked in orange which represents a consecutive success period. If these periods are detected consecutively within overlapping windows, these are consolidated and considered as a single period of consecutive successes with a longer length. Various parameters can be set to detect consecutive successes. A larger *N* indicates longer consecutive successes, while a smaller *N* means shorter ones. Instances detected with parameters like $X/N=5/9$ are regarded as strong hot streaks, and $X/N=1/1$ captures the weakest form of ones, which is a single success. The occurence timing of consecutive success is the starting position of consecutive *N* papars. The timing is normalized for different career lengths among researchers. For example, the relative timing of the *i*-th paper in a career is calculated as $i/(N_{T}-N)$, where $N_{T}$ is the total number of published papers.

The previous study captured consecutive success by using the hot-streak model^4^. The hot-streak model assumes a temporal rise in a researcher’s potential and thus is applied for the moving average of career sequence data with the window size of $\Delta N = max(5,0.1N_T)$. The size is ceiled by 10% of a career length to smooth out influence by random factors. However, we find that the window size could determine the duration of hot streaks (Supplementary information 3.3). The average duration of 3.7 years derived by the hot-streak model may be an artifact based on single success stretched by the window size, suggesting it may not accurately capture the true sequence of consecutive successes in a career. Contrarily, our approach more directly characterizes consecutive successes by examining raw career sequence data with fewer artificial assumptions.

Detected consecutive successes with several parameters show intriguing characteristics. Firstly, we found that these are less likely to occur in the mid-stage of a career but are more likely to appear in the early and late stages (Fig. 1b-d). Especially, single success are spread flat in the career, but consecutive successes concentrate on career beginnings and ends (Fig. 1b). Further, moderate successes, $k=50\%$ for example, happen randomly, but intensive successes like $k=5\%$ are more likely to happen in the early and later phases (Fig. 1c). Shuffling the order of career sequence data eliminates the U-shaped distribution, which means that the pattern of early and late career successes is not a coincidental phenomenon (Fig. 1d). These findings indicate that consecutive successes do not occur at a constant probability throughout a career. Instead, the likelihood of their occurrence is concentrated on the early and late stages, especially for successes of greater length and intensity.

Looking at the starting year distribution of our consecutive success extraction, the observed years of consecutive success tend to increase as careers progress into their later stages. However, compared to instances of single hits, the mid-career periods, from 7 years after the career begins to 5-10 years before the career ends, show lower likelihoods of achieving consecutive hits (Supplementary information 2.1). This illustrates the dynamics whereby true consecutive successes manifest initially during the Ph.D. and early postdoctoral phases, subsequently subside during the mid-career period, and become less likely until the final stages of one’s career. These U-shaped patterns demonstrate robustness over time (Supplementary information 2.2) and, even when considering variations in career dynamics of success across different fields, mostly hold true (Supplementary information 2.3). Our identification method shows U-shaped distributions over the career but the hot-streak model demonstrates that consecutive successes occur with consistent probability throughout careers^4^. We estimated that this discrepancy mainly comes from preprocessing for career sequence data and false positive detection of the previous method (Supplementary information 3.3).

## Patterns behind consecutive successes

To understand factors related to the occurrence of consecutive successes, we characterize the career-stage dependency of indicators related to team activities. Science and collaborations are closely intertwined, with factors such as team size^11^, the strength of connections between authors^9,16^, and the topics^10,18^ particularly influencing scientific outcomes. Based on these indicators, our findings revealed unique patterns associated with consecutive successes.

**Fig. 2:**
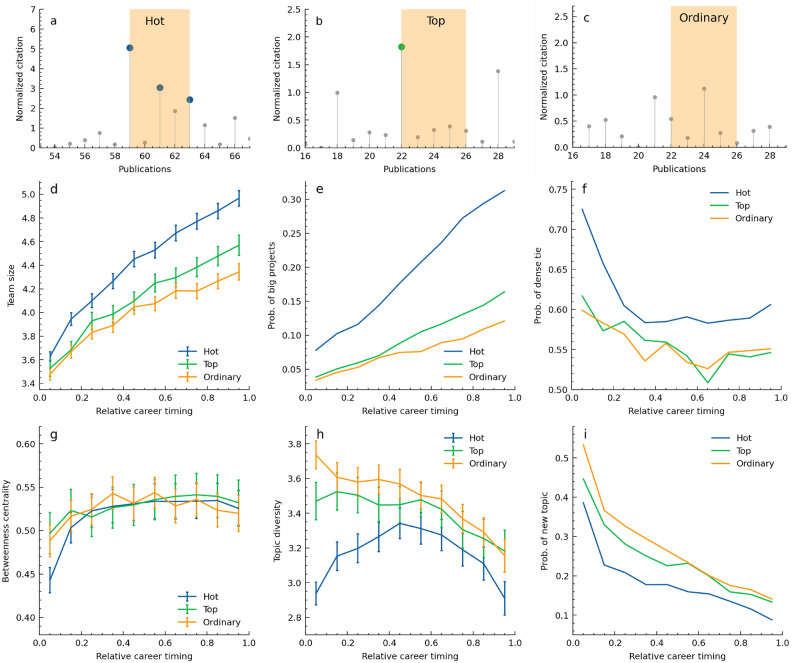
Characteristics of consecutive successes. (**a**) A typical example of a ‘Hot’ career sequence. The period filled in orange is detected as a hot streak of five consecutive papers. Blue dots represent top 10% papers, appearing three times within the five papers. (**b**) An example of ‘Top’ sequence, represents a period of single success, not consecutive. A green dot indicates a top 10% paper, appearing once among five papers. (**c**) ‘Ordinary’ sequence example as comparisons, five consecutive non-top 10% papers, representing a period without significant hits in careers. (**d**-**i**) Comparison of the 3 sequences across 6 metrics. The relative career timing is divided into ten bins, and metrics are calculated for sequences with the same start timing in each bin. This allows observation of how metrics vary in early, mid, and late career stages. (**d**) Team size, focused on author lists of fewer than 10 people (Supplementary information 4.1). (**e**) Proportion of teams with 10+ members. (**f**) Proportion of sequences with dense ties, appearing 3 or more times in the co-author list of the 5 consecutive papers. (**g**) Betweenness centrality of the focal author in the co-authorship network of the 5 papers. (**h**) The number of topics tackled in the 5 papers, ranging from 1 to 5. (**i**) Proportion of new topics not previously tackled. In (**d**) and (**f**-**i**), calculations are performed on sequences where the team size for all 5 papers is less than 10. The bars in (**d,g,h**) represent the 95% confidence interval that includes the estimated population mean, assuming a normal distribution. See Methods for details of definitions and calculations of each metric.

We defined three types of career sequences: ‘Hot’, ‘Top’, and ‘Ordinary’ (Fig. 2a-c). ‘Hot’ represents consecutive success with parameters of $X=3$, $N=5$, $k=10\%$, that is five consecutive papers by the same author including at least three highly-cited ones. ‘Top’ denotes single success, including five consecutive works starting with a top 10% paper but fewer than three high-impact papers. ‘Ordinary’ works comprise five non-top 10% papers. Our analysis covered a sufficient number of each across all career stages: 47,356 ‘Hot’, 25,227 ‘Top’, and 39,278 ‘Ordinary’ sequences (see Methods). This comparison helps us to uncover several patterns of ‘Hot’ career sequences.

1. **Large teams**: We found that larger teams are more common in later career stages and for ‘Hot’ sequences, suggesting that consecutive successes are more likely to arise from larger teams than ‘Top’, and ‘Ordinary’ (Fig. 2d), which is consistent with the previous study^5^. This trend is measured in teams of nine or fewer members.
2. **Big projects**: We defined teams of 10 or more as ’big projects’ and calculated the probability of such projects. If at least one paper in a consecutive period is a big project, the sequence is counted as a big project. The later career stages have a higher probability of big projects, with ‘Hot’ sequences notably more prevalent than the other two types (Fig. 2e), indicating that big projects are likely to be in a series of high-impact works of an individual’s career. The probability of ‘Hot’ sequences having papers with more than 100 authors is about ten times higher than the other two types, no matter the career stage (Fig. S15).
3. **Dense ties**: To understand collaboration patterns in depth, we analyzed co-authorship networks of five consecutive papers (Methods). Observing the presence of dense ties, defined as co-authoring three or more of the five papers with the same authors, we found that ‘Hot’ sequences consistently show a higher probability of dense connections throughout a career (Fig. 2f). We also hypothesized that authors with consecutive successes might have higher betweenness centrality in co-authorship networks, acting as hubs connecting different author groups. However, no significant overall difference is observed, suggesting that the network structure of collaborations doesn’t vary significantly (Fig. 2g). A higher collaboration rate with specific authors could contribute to experiencing successful periods.
4. **Topic focus**: The diversity of research topics in five consecutive papers is investigated (Methods). ‘Hot’ sequences generally exhibited less topic diversity, indicating a more focused approach and the pattern shows an inverse U-shape with a decrease in early stages, an increase in mid-career, and a decrease in later stages (Fig. 2h). The stronger focus in early and late stages might relate to the distribution of consecutive successes being more common in these phases.
5. **New topics**: We also examined the probability of new topics tackled during the sequences, which had not been addressed in the author’s previous career (Methods). In ‘Hot’ sequences, throughout a career, the likelihood of venturing into new topics is lower than in the other two types (Fig. 2i). This indicates that revisiting previously tackled research topics is related to consecutive success, providing hints for topic selection strategy.

Overall﻿, consecutive successes tend to occur in larger teams, with frequent collaboration with the same authors, focusing on fewer topics, and these topics are often previously addressed. These patterns suggest particular strategies and environments facilitating successive high-impact scientific works at different career stages.

## Early consecutive success from dense ties, later from large teams

What explains the U-shape distribution of consecutive successes along careers? In the early stages, we see small teams, dense ties, and a focus on research topics. These suggest that early-career researchers experience hot streaks with working intensively with a few co-authors, such as mentors and their group members. As careers progress to the middle stage, the number of collaborators increases, the probability of working with specific individuals decreases, and topic diversity grows. This trend might reflect the expanding collaborator network over time. In later stages, team size grows further and researchers tend to focus on specific research topics. The established reputation and authority of senior scientists might enable them to steer large teams toward focused topics.

**Fig. 3:**
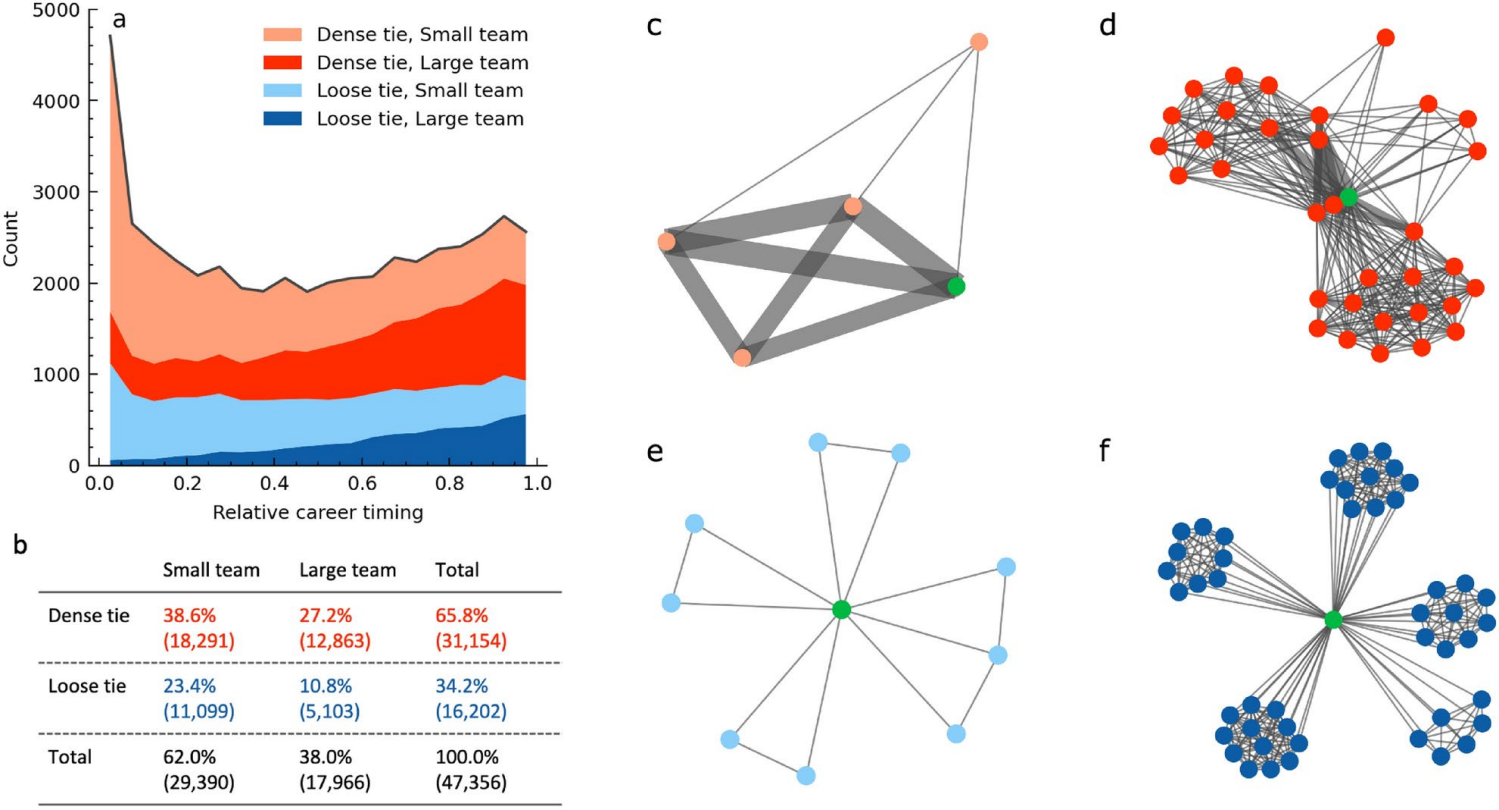
Four types of consecutive successes. (**a**) The histogram when consecutive successes occur with parameters $X/N=3/5$ and $k=10\%$. The four types of them are displayed in a stacked manner, each represented by a different color. The top two types, shown in pink and red, represent consecutive successes with dense ties, more common in early and late stages. The bottom two, in light blue and blue, depict loose ties occurring consistently across the career. (**b**) The four types’ proportions. 65% of them originated from dense ties and 40% originated from large teams. (**c**-**f**) Typical co-authorship networks for each type. Green nodes represent the focal author, other nodes are co-authors, and edges indicate the number of collaborations in five consecutive papers, ranging from one to five. (**c**) Dense ties in small teams are shown as thick edges between a few nodes. (**d**) Thick edges with large teams composed of many co-authors. (**e**) The focal author is connected with fewer authors and thinner edges representing loose ties and small teams. (**f**) No thick edges with many co-authors show collaboration in loose ties with large teams.

Given the distributions of consecutive successes involving dense ties and big projects (Fig. 2e, f), we find that these two factors contribute to the U-shape distribution shown in Figure 1b. Consecutive successes within loose ties, which do not involve as frequent collaboration as dense ties, occur with almost constant probability throughout a career, while those involving dense ties contribute to the spike at the early stages and the slight increase in the later stages (Fig. 3a). As careers progress into the later stages, the proportion of consecutive success originating in small teams decreases, while those arising from larger teams increase. Hereinafter, small teams mean less than 10 members and large teams mean ten or more, i.e. big projects. These indicate that both early and late career successes are dominated by dense ties, while later stages see stronger dominance by larger teams. Thus, by considering two factors, the strength of connections and team size, we can categorize types of consecutive successes into four distinct groups. Notably, about 40% of consecutive successes originate from teams of more than ten members (Fig. 3b). Each type is intuitively understood by visualizing their co-authorship networks (Fig. 3c-f).

Dense ties and large teams can explain why mid-career scientists less frequently experience consecutive successes. During mid-career, dense connections are fewer than in the early stages, and the team size is not sufficiently large. This situation could make consecutive successes more challenging.

**Fig. 4:**
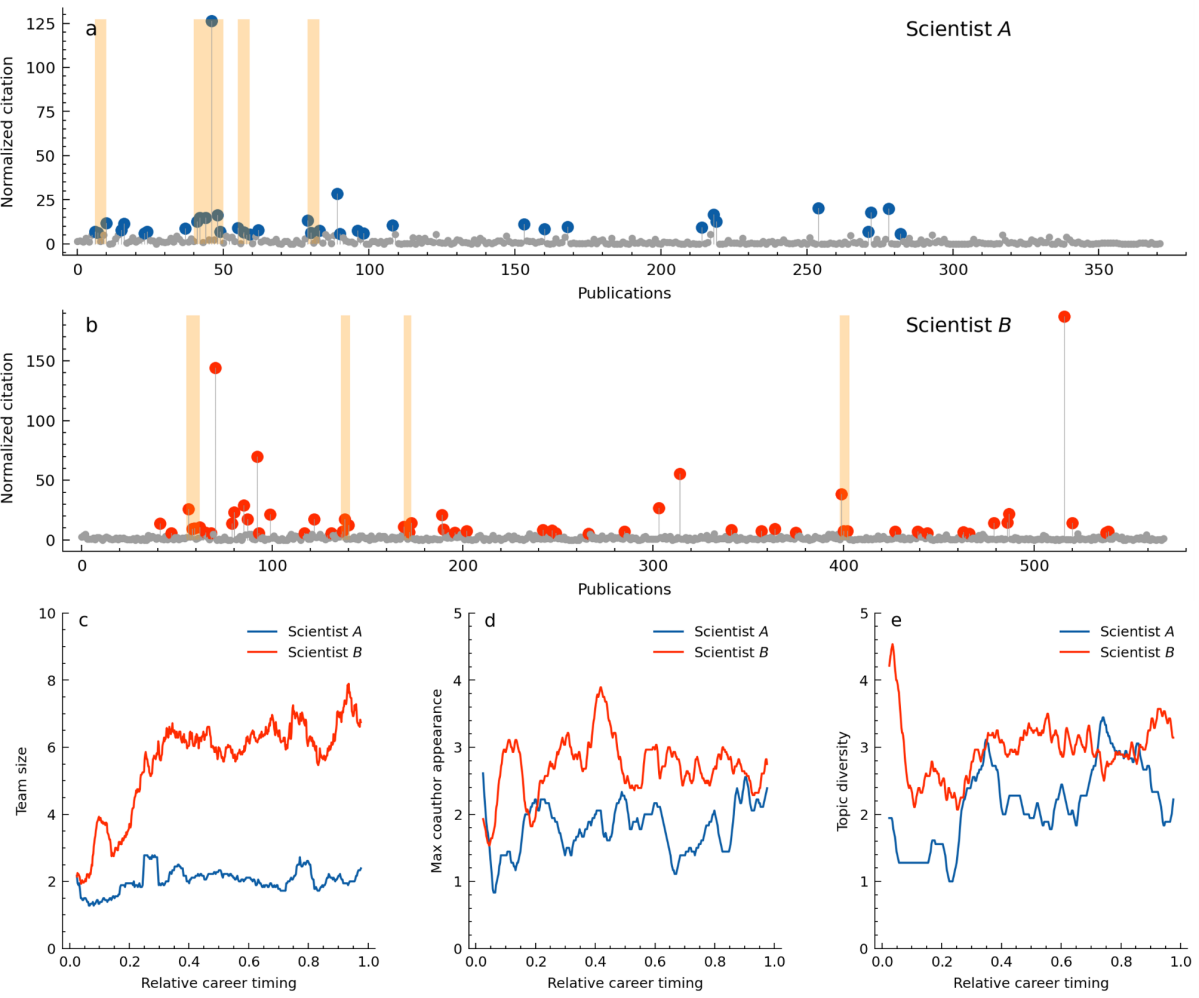
Two scientists’ typical career dynamics. (**a**,**b**) Career sequences. The papers with top-10% impact are colored and the orange areas indicate consecutive success identified by our method. (**c**) Relation between papers’ publication timing in normalized career length and their team size for two scientists. (**d**) Frequency of co-authors in five consecutive papers; higher values mean repeated collaborations with the same authors, reflecting dense ties. (**e**) Topic diversity dependency on career stages, measured by the variety of topics in five consecutive papers. For clarity, (**c**-**e**) display moving averages with a window size of 5% career length.

As examples, we consider two physicists in condensed matter physics. The field has received significant contributions from Japanese scientists, including some renowned researchers. Scientist *A* made significant theoritical contributions and Scientist *B* is a great experimental researcher. Both have produced over 350 papers throughout their careers, showing their high productivity. Scientist *A* produced high-impact works early in his career, going through four consecutive successes (Fig. 4a), with his most significant work included in his second one. On the other hand, Scientist *B* had three consecutive successes early in his career and then another later on (Fig. 4b). Tracing their work trajectories revealed interesting differences. Upon normalizing the length of their careers and comparing team size, collaboration density, and topic diversity, it was found that Scientist *B* worked with larger teams later in his career (Fig. 4c). The maximum co-author frequency and topic diversity across five papers tend to be higher for Scientist *B* throughout his career, but the difference is relatively small (Fig. 4d,e). Indeed, in the later stages of his career, Scientist *B* produced his most significant paper in graphene, outperforming his early discoveries, with over 10 co-authors. Such a pattern of consecutive success with larger team sizes in the later career aligns with the overall trend observed among scientists (Fig. 2d).

## Discussions

In this study, we hypothesized that the mechanisms underlying consecutive successes vary depending on career stages and thus focused on analysis from the perspective of collaboration patterns. We found that the hot-streak model^4^ may not always capture consecutive successes, i.e. hot streaks because there’s a possibility that single success may be mistakenly identified as consecutive successes (Supplementary information 3.3). By extracting consecutive successes by more directly observing sequences of highly-cited works, we found that a series of successes is more likely to happen in the early and later stages of a career, with a dip during mid-career. Early-career hot streaks are more likely among researchers with dense ties to specific colleagues, whereas late-career hot streaks are often supported by big projects on familiar research topics. The uncovered empirical regularities highlight the importance of considering scientists’ successes at each career stages. Early in the career, dense connections are present, but as the career progresses and the academic network expands forward, larger team sizes emerge, showing a complementary relationship. The “mid-career pitfall” may be attributed to the loss of these two factors.

The prevalence of hot streaks in the early career suggests a potential survivorship bias in academia. Our study focuses on researchers with at least 30 publications over a 20-year period. Such those who have long and successful careers in academia likely survive due to excel of their early successes. The U-shaped pattern is robust as evidenced by the fact that the pattern remains even when the 20-year career condition is removed and only researchers with over 30 papers are considered. Yet at the same time, this analysis still excludes individuals, e.g. who wrote only a few papers in their early career and then left academia. The impact of their work or the strength of their ties is not investigated, implying that our approach might overlook hidden talents and collaboration. Analyzing hot streaks while considering individuals who left academia early, as well as investigating the causal impact of early and late career trends, will be crucial tasks for future research.

The emergence of hot streaks among researchers with dense ties in the early stages implies that survival as a scientist may depend not only on individual potential but also on the chance of forming a hot streak through good mentorship initially. In this respect, our empirical results are associated with prior research on the positive effect of connecting with top researchers early in one’s career^12^. This type of tie likely affects the magnitude of later success and sustaining a research career.

The divergence of success distribution due to exploration, followed by a re-concentration towards the end of a career, is highly suggestive. Researchers in their later career stages shine by forming large teams of more than ten people and refocusing on familiar topics. This is particularly evident in experimental fields that can expand the scale of experiments with significant budgets, such as Health Science and Manufacturing Engineering, where many hot streaks are experienced in the last 10% of a career (Fig. S4). In contrast, fields emphasizing theory, like Algebraic Geometry and Network Science, experience fewer late hot streaks. This disparity reflects the relationship between funding strategy and fields where costs and outcomes are more or less directly proportional. In experimental fields, forming large teams can lead to hot streaks at any age. Or, the single success, which can occur at any point in a career, may be elevated to consecutive successes due to the social capital that senior researchers possess, such as their connections with other researchers and the trust they have earned within the academic community. Large teams may serve as a proxy variable for such recognition by the research community, but establishing the causal relationship will be a matter for future work.

The mid-career, spanning from the 10th to 20th year, is challenging. Increased independence and responsibility, departures from initial support structures, and the expansion of administrative tasks requiring multitasking make it difficult for researchers to focus on specific topics and allocate resources. Researchers not only move between organizations seeking positions^20^ but also belong to more research communities, frequently switching topics^10^. These career dynamics increase the cost of adapting to new environments. In addition to these factors, a decline in the number of publications in mid-career^21^, looks influence mid-career dip of consecutive success. The discontinuous success in mid-career may be due to limited time, human resources, and the difficulty of returning to familiar settings. Yet, in the later stages, forming large teams can contribute to a revival of hot streaks. Taken together, if a stable research foundation is established earlier in the mid-career, it would lead to greater achievements, which could further accelerate scientific progress. It is necessary to take measures to put mid-career researchers in the spotlight.

The reality of mid-career researchers’ support is also reflected in national research support policies. For example, in Japanese funding systems, there are opportunities available to all researchers and those specifically tailored to support young researchers. However, few fundings specifically targeted mid-career researchers^22^. The system has been improved so far, but it still lacks clear mention of support for mid-career researchers. Consequently, mid-career researchers are forced to compete with more experienced researchers for funding, potentially influencing the occurrence of hot streaks. Single success can occur randomly at any career stage, suggesting that such kind of success can be achieved even without funding, but sustaining successes and maximizing output on specific topics might require new funding strategies targeted at the overlooked mid-career researchers.

One of the clues to understanding them is that they might be taking risks by exploring new ideas while the possibility of achieving consective successes is relatively lower. The topic diversity of hot streaks is higher in mid-career compared to early and late stages (Fig. 2h). This suggests that they are deviating from previous research topics to explore new ideas, and the works produced might be different. Indeed, we found that papers in hot streaks of the middle or late career stages tend to have higher disruptiveness rank than papers in the early stages (Fig. S16). The dense connections that characterize early hot streaks highlight the importance of networks, possibly supporting tactics that help scientists increase citations within their community. In contrast, mid-career researchers may move away from such tactic behavior, taking on riskier projects instead. It may be essential to provide support that encourages their challenges and fosters disruptive innovation.

Our study has limitations common to this style of analysis. Firstly, the results may be biased due to the dataset used. Since Scopus is limited to reputable journals designated by Elsevier, the observed consecutive successes are confined to works of a certain level of renown. By restricting data to the years 1970 to 2012, the dataset may not fully capture the beginning or end of the careers of individuals who started their careers before 1970 or whose careers continued beyond 2012 (Supplementary information 1.2). Secondly, while this study focuses on common trends in how collaboration patterns change during consecutive successes across careers, there also is the diversity across disciplines (Fig. S4). The U-shaped pattern does not appear universally across all fields, indicating that a more detailed analysis on the content of the disciplines is necessary in the future to understand what determines the occurrence of hot streaks. The patterns discovered in this study specifically target only academic careers and may not directly apply to a wider range of creative fields, such as artists, film directors, musicians, or ballet dancers^5,23,24^. Lastly, the findings of this study represent correlations rather than causations. While it is true that dense ties in the early stages and larger teams in the later stages are commonly observed during consecutive successes, real experiments are required to determine whether interventions can lead to the occurrence of hot streaks.

For future research, there is a need to investigate the broader effects of hot streaks within the overarching framework of scientific mechanisms, moving beyond mere observation of consecutive success. While our study has shed light on the interplay between dense ties and consecutive successes, there might be a potential exacerbation of researcher fragmentation if individuals persist within narrow disciplinary confines. Furthermore, while hot streaks may yield significant impacts, they could inadvertently limit the cultivation of diverse perspectives through collaboration, potentially leading to a narrowing of research topics in later career stages. Comprehensive analyses are needed to assess the multifaceted impacts of consecutive successes on scientific progress, including their effects on collaborative dynamics and the emergence of subsequent research.

## Methods

### Detection of consecutive successes

Given an author $a$, the set of papers published by this author is denoted as $P_a = \{p_1, p_2, ..., p_{N_T}\}$, where $N_T$ is the total number of published papers. The 10-year normalized citation count for a paper $p$ is represented by $C_{10}^p$. The subset of papers $P_a^{\text {k}} \subset P_a$ includes papers whose $C_{10}^p$ are in the top $k\%$ of all $C_{10}^p$ in $P_a$. For a specified window $N$, a time interval from $i$ to $i+N$ is considered as a consequtive success if:

1 $$\begin{aligned} \left| \{p \in P_a^{\text {k}}\ |\ p \in p_i, ..., p_{i+N} \}\right| \ge X \end{aligned}$$

Here, $|\cdot |$ denotes the cardinality of a set. Eq. (1) indicates that if the number of papers in $P_a^{\text {k}}$ published within the window from $i$ to $i+N$ is more than $X$, the interval to be considered consequtive success. This method detects overlapping periods in a career sequence, and we have incorporated a process to merge such instances. As a result, the length of consecutive success is usually five papers, but it can be longer. The number of people experiencing hot streaks and the frequency of these occurrences vary depending on the definition (Fig. 1b-c). Compared with previous hot-streak model^4,5^, which capture the potential change of reseacher’s output, this definition is more data-centric approach focusing on the actual output of researchers.

Some authors experience more than one hot streak during their careers. Under the conditions of $X = 3, N = 5, k = 10\%$, this proportion is 13%. We also found that authors who produce more papers over their careers tend to experience hot streaks more frequently, and those who have hot streaks generally have a higher overall career impact. When handling career sequence data, we use the number of publications instead of years to normalize career patterns that vary across fields due to different publication frequencies and numbers^6^.

### Three types of sequences

To compare patterns of consecutive success across different career stages, we defined ‘Hot’, ‘Top’, and ‘Ordinary’ sequences (Fig. 2a-c). To determine these, first, we identified 47,356 hot streaks from 35,495 authors with the parameters $X = 3$, $N = 5$, and $k = 10\%$. Some hot streaks were longer than five papers due to merging processes, but only the first five papers were selected for ‘Hot’ sequences to align with the length of ‘Top’ and ‘Ordinary’. Next, for the 64,505 authors without hot streaks, we randomly chose five consecutive papers from their career data. If one or two of five papers were in the top 10%, the sequence was classified as ‘Top’, resulting in 25,227 cases. Otherwise, it was categorized as ‘Ordinary’, accounting for 39,278 cases. Each sequence was tagged with the relative publication timing of its first paper, scaled between 0 and 1, to analyze within the same career stages. Various metrics were then calculated, based on the information from these five papers, such as author lists and topics.

### Team sizes

For the dataset used in this study, which includes 9,476,817 papers, the average number of authors per paper is 28, with a median of 4, a minimum of 1, and a maximum of 3,220. Notably, 88% of the teams consist of fewer than ten members. Considering previous study^11^ and computation time, the characterisation in Figure 2 were performed for sequences with all five papers having fewer than ten authors each. We used a team size of ten as a benchmark for defining team sizes. ‘Big Project’ is defined as a sequence of five papers where at least one paper includes more than ten authors (Fig. 2e). The classification of whether a consecutive success involves large teams is based on whether at least one of the papers has more than ten authors (Fig. 3a).

### Dense ties and loose ties

To evaluate the collaboration patterns of a focal author’s five consecutive papers, we examined their co-author list. If a co-author appears in all five papers, it signifies a strong collaboration, giving a maximum co-author appearance of five. If each paper has a different team without any overlaps, the value is one. We define sequences with a co-author in at least three out of five papers as having ‘dense ties’.

### Topic diversity

The research topics of papers are calculated from co-citation network clustering of each author’s publication^10^, applying modularity maximization by Louvain method with resolution $=$ 1. For each consecutive paper, a topic diversity score is defined as the number of topics in these papers. The score is five when all papers are on different topics, while a score of one indicates all papers share the same topic. This score reflects the focal author’s focus on a single or multiple research topics during that period. The topic of each paper is calculated from clustering co-citation network on each researcher’s publications^10^ (Supplementary information 1.1).

### New topics

Within the targeted five papers sequence, we identify the most frequent topic $T_f$. If there is a tie for the highest frequency, the first topic appearing in the sequence is $T_f$. If $T_f$ does not exist in the author’s earlier career, the attribute ‘New Topic’ is true; if it was present before, it is False.

## Supplementary Information

Supplementary Information.

## Data Availability

The data that support the findings of this study are available from Elsevier but restrictions apply to the availability of these data, which were used under license for the current study, and so are not publicly available. Data are however available from the authors upon reasonable request and with permission of Elsevier. Data requests will be handled by the corresponding author, Noriyuki Higashide
