# Supplementary material for: Mid-career pitfall of consecutive success in science
Source: Sci Rep. 2024 Nov 15;14:28172. doi: 10.1038/s41598-024-77206-y (PMC11568327; doi:10.1038/s41598-024-77206-y)
Supplement: Supplementary file 1 — Supplementary Information. [file 41598_2024_77206_MOESM1_ESM.pdf]

Supplementary information: Mid-career pitfall of consecutive success in science

Noriyuki Higashide[1,*], Takahiro Miura[1], Yuta Tomokiyo[1], Kimitaka Asatani[1], and Ichiro Sakata[1]

[1]Department of Technology Management for Innovation, Graduate School of Engineering, The University of Tokyo, Tokyo 113-8656, Japan
[*]Corresponding author, n.higashide@ipr-ctr.t.u-tokyo.ac.jp

Contents

1 Data description **1**
1.1 Career sequence data . . . 1
1.2 Potential limitation of datasets . . . 2
2 Probability of consecutive success **3**
2.1 Year distribution of U-shape success . . . 3
2.2 Time dependency of U-shape success . . . 3
2.3 Field dependency of U-shape success . . . 4
3 Relation with the existing model **6**
3.1 Empirical measurements . . . 6
3.2 Revisiting hot-streak model . . . 7
3.3 Dependency of the hot-streak model on window size of $\Gamma(N)$ . . . 9
3.4 The hot-streak model and our data-centric detection . . . 9
4 Collaboration patterns **13**
4.1 Team sizes . . . 13
4.2 Examining if early and late hot streaks come from the same work . . . 13
4.3 Disruption index of hot works and works of who experience hot streak . . . 13

1 Data description

1.1 Career sequence data

We prepared a home-built dataset of research papers based on Elsevier's Scopus Custom Data, encompassing all documents recorded between 1970 and 2021. As the most comprehensive database for abstracts and citations of peer-reviewed articles, Scopus contains over 73 million papers and 1.2 billion citations across a broad range of research fields and hence frequently utilized by researchers for bibliometric and citation analysis[1–3]. We focused on researchers who had sufficiently long careers to characterize timely-clustered success in life-long careers. Hence, we randomly extracted 100,000 authors who published more than 30 papers and 20 years of their whole careers from the dataset. To calculate citations within 10 years of publication, papers published from 2013 to 2021 are removed from the author information. The historical order of publications was organized on a monthly basis, according to their publication dates.

To assess each paper's impact, we measured its citations within 10 years of publication, denoted as C_{10}[4]. We then adjusted for field-specific and annual variations by dividing it by the average citations for that field and year[5]. To obtain field information at the publication level, this study clustered the whole citation network in 2021 using the Leiden algorithm based on the Constants Potts Model ($\gamma = 10^{-6}$)[6,7]. Clusters with a size of less than 10^5 papers were merged into other clusters with the strongest citation connections[8], resulting in the identification of 66 distinct fields. This re-scaling accounts for calibrating publication frequency and impacts across disciplines.

Each paper's topic is calculated by clustering the co-citation network within each career[9]. The topics were calculated by partitioning the co-citation network of papers published by each scientist using the Louvain method with a resolution parameter of $\gamma = 1$.

1.2 Potential limitation of datasets

The dataset used in this study has several limitations. Firstly, this research targets researchers who have published 30 or more papers over a span of more than 20 years in Scopus, which may introduce survivorship bias and publication bias. For instance, researchers in fields such as law and philosophy, who may spend years producing a single book or publication, are less likely to be included in the dataset. Additionally, a plausible hypothesis that researchers who do not experience a hot streak may exit their careers earlier cannot be tested with this study. Moreover, publications in languages other than English are not recorded, which means that different outputs in other languages during periods of consecutive success might not be captured. Scopus, by incorporating not only articles and conference proceedings but also book chapters, editorials, and letters, necessitates the consideration of diverse patterns of success that may vary according to document type.

Secondly, in the analysis of teams, the size may appear larger than in reality due to Elsevier's consolidation of group authors, which unfolds group contributions into individual ones. Group authors, such as the "CONSORT Group," "AHA Heart Failure Taskforce," or "XY Workshop Members," attribute contributions to a group rather than individuals for outputs resulting from specific activities. Since the number of collaborators in such groups can be quite large, often in the dozens, this could potentially affect the perceived team size and density of ties.

Thirdly, although this analysis categorizes career stages as early-career and late-career within the dataset, the focus on publications from 1970 to 2012 means that individuals who began their careers before or continued their careers after this period might not have their actual career lengths accurately represented.

2 Probability of consecutive success

2.1 Year distribution of U-shape success

Although we have demonstrated the presence of a pitfall of consecutive successes in the mid-career phase on a relative career basis, it remains to be determined at what specific year of their career researchers are most susceptible to this phenomenon. Figure S1 illustrates the timing of consecutive success occurrences extracted using our model, translated into periods within a researcher's career. Researchers were divided into five groups based on career year length, and the distribution of single and consecutive successes was shown for each. It was found that consecutive successes become less likely approximately from seven years after the career begins to 5-10 years before the career ends. These timings likely correspond to the end of youth, the later stages of postdoctoral, and the periods heading towards retirement to complete careers. This indicates that researchers in their mid-careers may lack sufficient resources for adequate exploration and exploitation. Note that the continuous increase in the number of single hits, which remain flat in terms of relative career from the early to the late stages, suggests that the number of publications tends to increase over time, possibly due to the utilization of collaborations, among other factors.

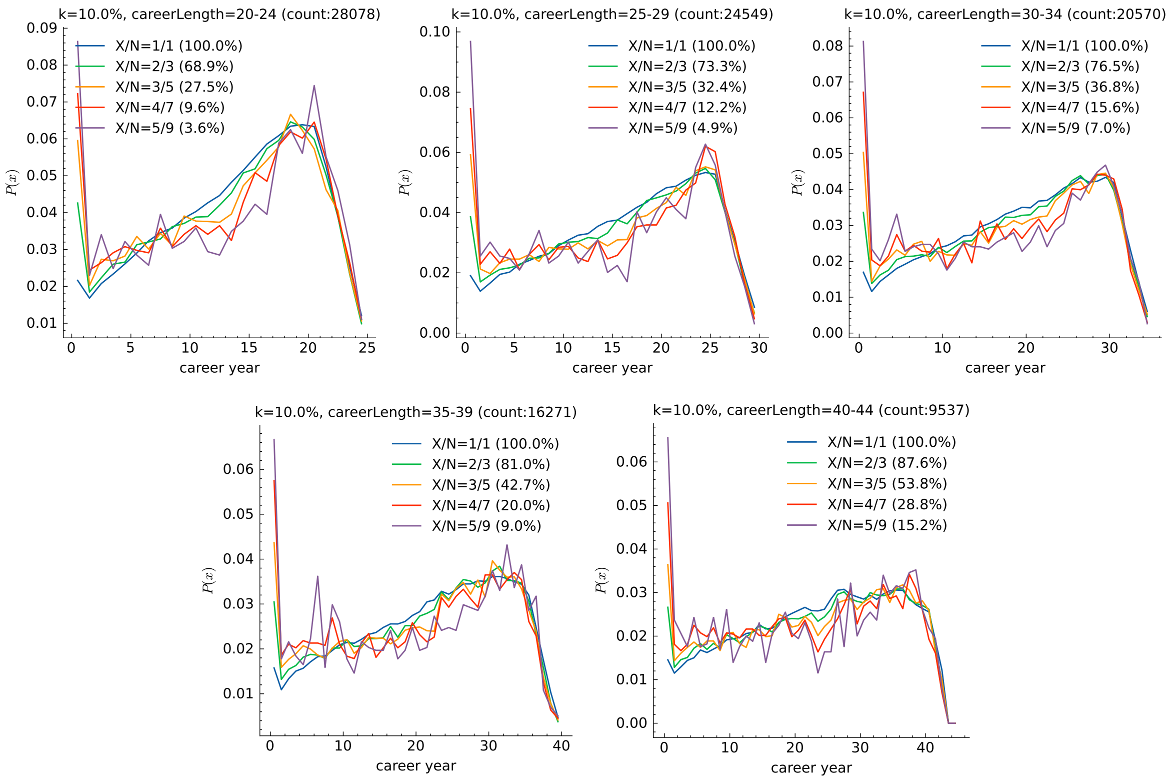

Figure S1. The distribution of consecutive success among researchers with varying career lengths in five-year increments. Regardless of age, the probability of consecutive success declines from seven years after the career begins to 5-10 years before the career ends.

2.2 Time dependency of U-shape success

The U-shape we observed might not just be about successes at the start or end of a scientist's career. Alterations in the dynamics of science can also serve as a potential confounder. For example, if it is easier to publish high-impact papers around 2010 because of citation inflation[10], scientists at any time in their careers could have hot streaks recently. To check this, we split scientists into five groups based on when they started their careers and how long their careers were. Then we checked if the U-shape still showed up.

We found that no matter when a researcher started their career (Figure S2) or how long they've been working (Figure S3), their single big successes happen randomly, but their hot streaks follow a U-shaped pattern. This is

especially true for researchers with longer careers, as they publish more papers and thus have more chances for these hot streaks. For example, out of 9,537 researchers who have been publishing for over 40 years, 5,131 (about 53.8%) had a hot streak where 3 out of 5 of their papers were in the top 10%. This shows that the U-shaped pattern happens no matter the time period.

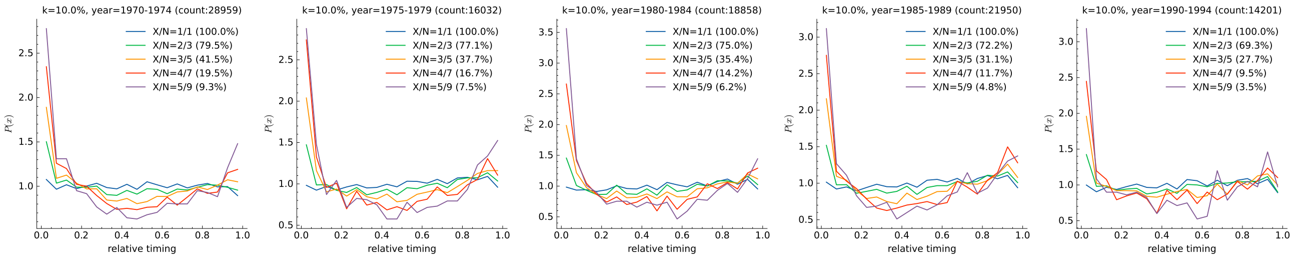

Figure S2. The distribution of hot streaks among researchers with varying initiation periods of career in five years increments. Regardless of the ages, the pattern of consecutive success delineates a U-shaped curve.

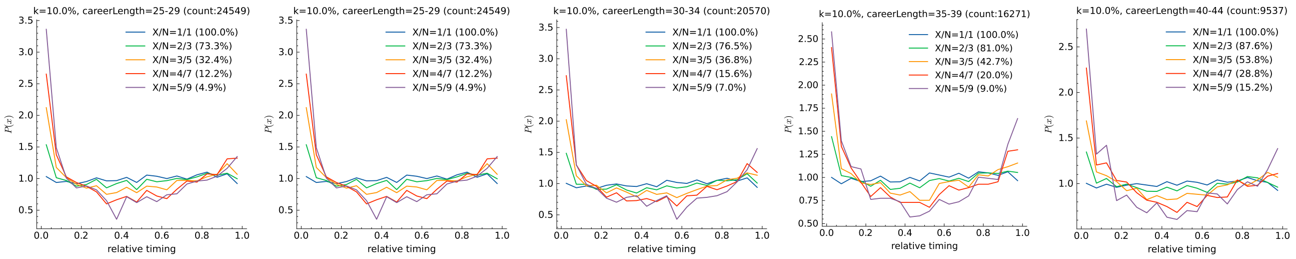

Figure S3. The distribution of hot streaks among researchers with varying lengths of career in five-year increments. Researchers with careers of any duration exhibit a U-shaped curve in their pattern of consecutive success.

2.3 Field dependency of U-shape success

In scientific contribution, there are crudely two types of studies: empirical and theoretical. Theoretical successes tend to appear early in a career, while empirical ones are more common later[11]. To make sure the U-shaped pattern isn't just showing these different types of scientific contributions, we looked at whether the distribution of hot streaks varied across 53 fields when we identified the most common topic during each hot streak as the topic of that streak.

We made a scatter plot in Figure S4. It shows if the probability distribution of hot streaks in each field is more than 1 either early in the career (relative timing below 0.1) or late (above 0.9). Out of 45 fields where more than 100 people experienced a hot streak (defined as k=10, X=3, N=5), 21 fields showed a U-shaped pattern with both early and late hot streaks. However, 18 fields had more early but fewer late hot streaks, like in Algebraic Geometry or Semiconductor materials and devices, which include both theoretical and empirical studies. Overall, early career hot streaks are robust, but whether one experiences late career hot streaks seems to depend on the field.

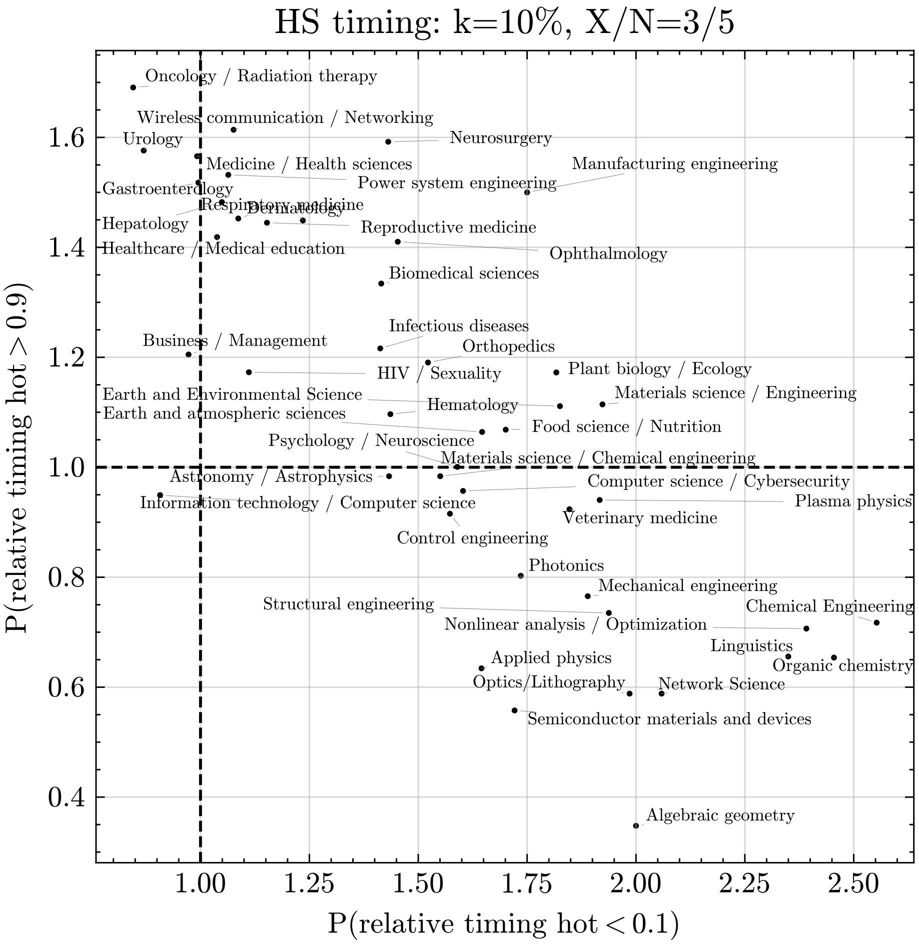

Figure S4. The distribution of U-shaped patterns in success across various fields (defined by k=10%, X/N=3/5). Each hot streak(HS) is categorized by its most common topic, and the graph plots the proportion of hot streaks occurring either early (probability P(x)<0.1) or late (probability P(x)>0.9) in a career. The thick dotted line indicates P(x)=1, with fields located in the upper right quadrant demonstrating a U-shaped pattern, signifying a higher occurrence of both early and late career peaks in success. The names of the fields were assigned based on the highest TF-IDF values of keywords in papers.

3 Relation with the existing model

3.1 Empirical measurements

Hot streaks are a phenomenon in which high-impact papers are concentrated at a time throughout a scientist's career[12]. Although there are minor data differences, we were able to confirm that the hot streak phenomenon is reproduced in our Scopus dataset.

We began by investigating the timing of the five most impactful works produced in each career. As confirmed in the original paper, we confirmed that the probabilities of when the top-impact works occur to each other are correlated. The normalized joint probability of the highest and second highest-impact work is calculated and displayed as a heatmap. Similar calculations were performed for each of the top 1-5 pairs and 10 combinations, and for all pairs, a pattern of high joint probability was observed on the diagonal (Fig. S5). This indicates that the timing of the appearance of the top 1-5 in the careers is correlated and that they tend to occur consecutively. This diagonal line disappears when the order of the carrier series data is shuffled, indicating that this tendency is unique to the actual data (Fig. S6).

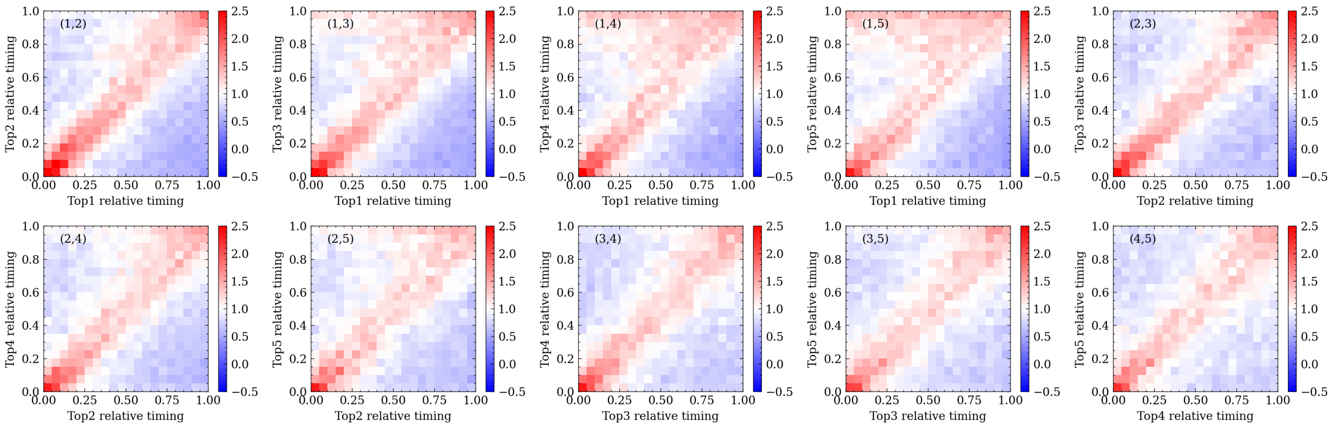

Figure S5. Distribution of normalized joint probabilities $P(x_1,x_2)/P(x_1)P(x_2)$ for the top-1 and 2 high-impact work, $x1$ and $x2$. Plotted 10 combinations of top 1-5 with the bins size of 20. The diagonal pattern indicates that hits tend to occur relatively consecutively. Relative timing is the Nth paper the researcher has published divided by the total number N_T. For example, the timing of the 8th paper published by an author who has written 80 papers would be 0.1.

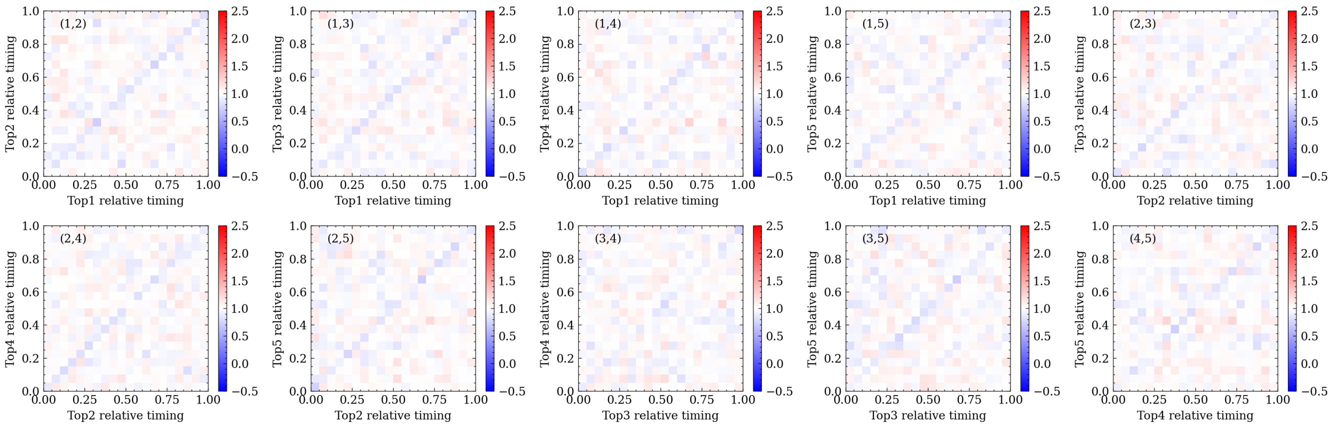

Figure S6. Distribution of normalized joint probabilities of the $x1$ and $x2$ for the career sequence data shuffled in order. The diagonal pattern has disappeared.

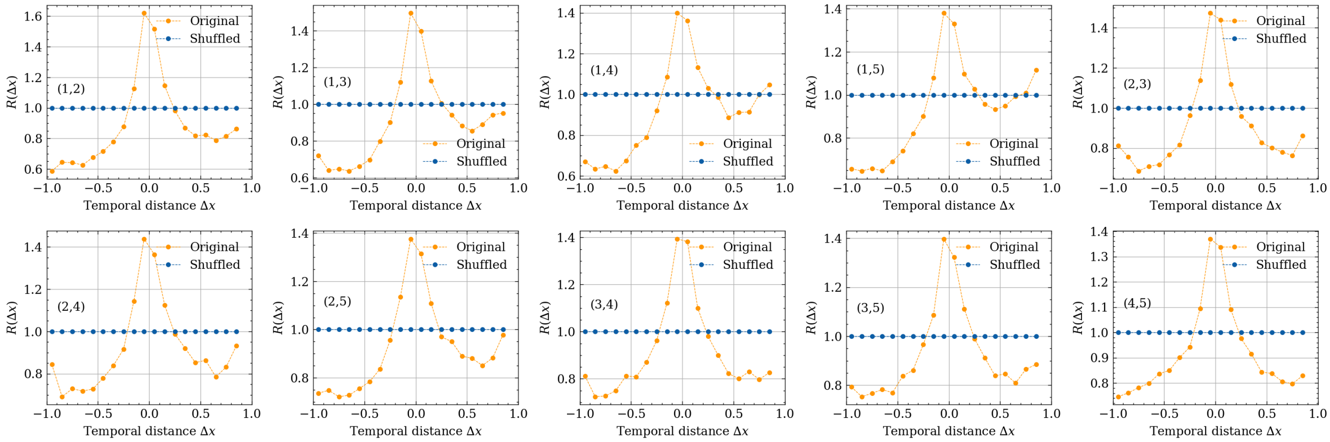

Figure S7. Normalized distribution of the difference in the relative timing of the top 1,2,3,4,5 papers. All are divided by the distribution of shuffled data. The yellow dots show the actual data and the blue ones show the shuffled data.

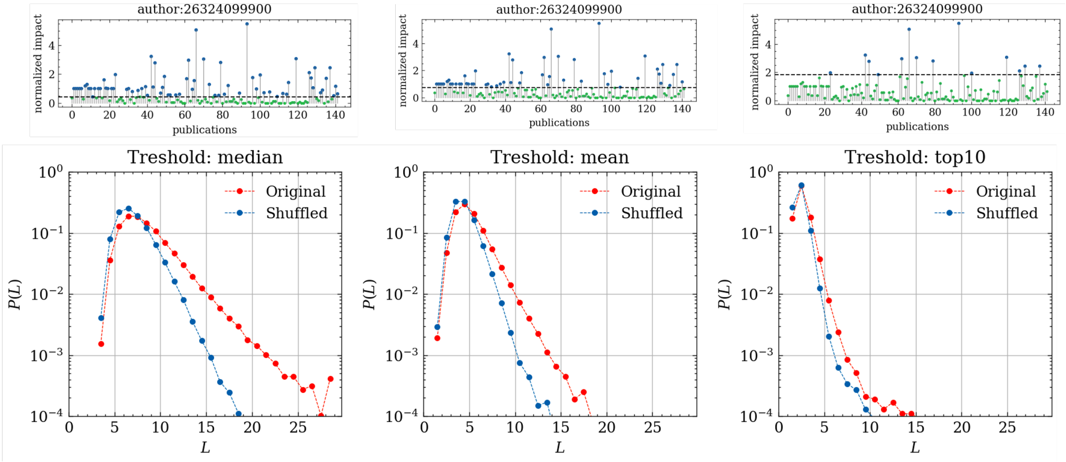

Figure S8. (Top row) Career sequence data for a scientist. The dotted line is the threshold, and higher-impact jobs are indicated by blue dots. From left to right: median, average, and top 10% thresholds. (Bottom row) Of works higher than the thresholds, the largest consecutive length is L, and its distribution is shown in red. Blue is for shuffled data. The higher the threshold, the shorter the L goes, but all of them are longer than the shuffle.

We defined temporal distance Δx as the difference in the relative timing of the top 1-5 papers to appear and divided the distribution $P(\Delta x)$ by the distribution of the shuffled data $P_s(\Delta x)$, we observed distribution $R(\Delta x) = P(\Delta x)/P_s(\Delta x)$ (Fig. S7). If $R(\Delta x)$ is higher than 1, it is more likely to occur than in the random case. The top 1-5 hits all have high values around 0 confirming that they are also more likely to occur at near time.

We replicated the original paper not only for timing but also for the length that high-impact works continue. We set a certain threshold and observed the distribution of the maximum length L, of the consecutive periods of higher-impact work, which tends to be longer than that of the shuffled data (Fig. S8). This indicates that the actual data tends to be more likely to be followed by a series of higher-impact papers than the shuffled data. To this end, hot streaks where high-impact papers are consecutive in time are also observed in our Scopus data set.

3.2 Revisiting hot-streak model

To explain the observed temporal regularities in career sequence data, the previous research has proposed a simple model[12]. First, they introduced a null model, where each work is randomly selected from a normal distribution

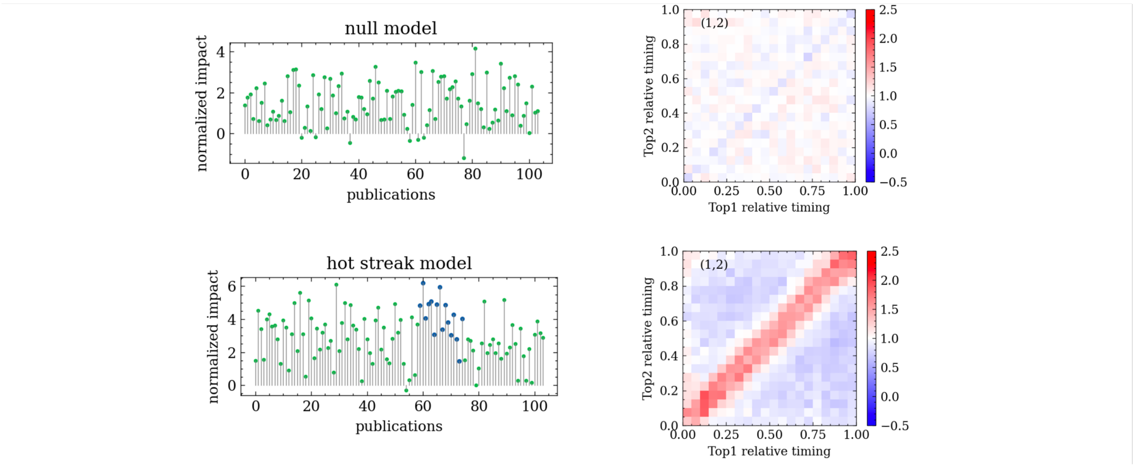

Figure S9. We generated two types of sequence datasets following the null model and hot-streak model respectively. As shown in previous research, we set Γ_0 to represent the average impact level of each researcher, with $\sigma) = 1.0$, and τ_H as 4 years.

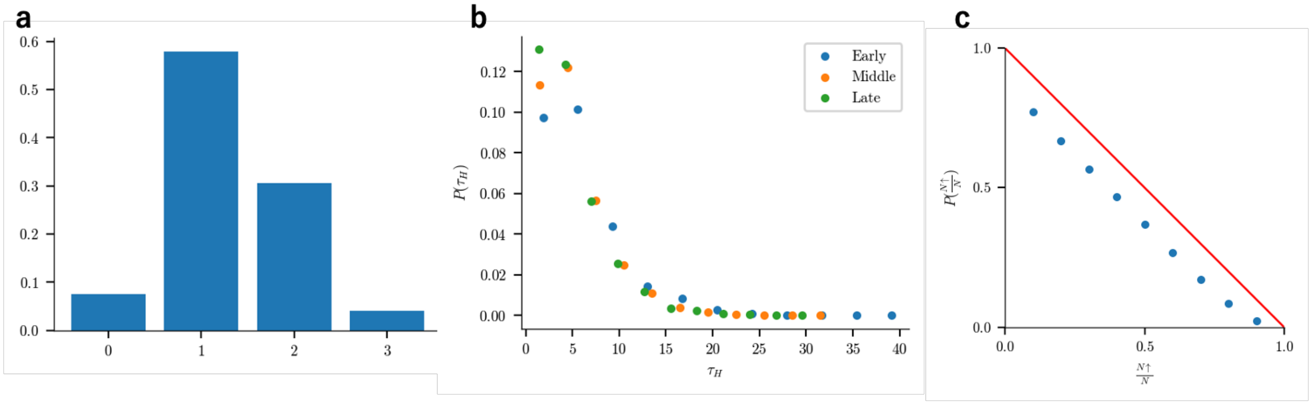

Figure S10. Characteristics of reproduced hot-streak model. **a**, Histogram of the number of hot streaks in a career. **b**, The distributions of durations of hot streaks $P(\tau_H)$. **c**, The cumulative distributions of the onset timing $P(N_\uparrow/N)$.

with parameters $\mathcal{N}(\Gamma_0, \sigma)$, where Γ_0 represents the individual researcher's specific parameter of impact level, and σ represents the variability in impact. With these two parameters and the total number of papers N_T, we can generate career sequence data following the null model. Next, they proposed hot-streak model, where Γ_0 increases to $\Gamma_H = \Gamma_0 + 1.0$ during a certain period τ_H randomly chosen within the career. By considering this simple assumption, they could reproduce patterns in actual data.

Using our dataset, we generated 100,000 sequence data following both the null model and the hot-streak model (Fig. S9). Interestingly, in the null model, the observed patterns in the simultaneous probability map did not exhibit diagonal patterns, while in the hot-streak model, diagonal patterns were observed. This indicates that the simple assumption reproduced the temporal proximity of success in the actual data, as reported in the prior study.

To apply the hot-streak model to actual data, we fitted a specific piecewise function to the moving average $\Gamma(N)$ of the career sequence data. This function resembled a square wave pulse and could have up to three rising edges, characterized by ten parameters:*base*, hot_1, $N_{1\uparrow}$, $N_{1\downarrow}$, hot_2, $N_{2\uparrow}$, $N_{2\downarrow}$, hot_3, $N_{3\uparrow}$, $N_{3\downarrow}$. Here, consistent with the previous research, $\Gamma(N)$ denotes the moving average over a window size ΔN, determined as 10% of the total number of works created, N_T on an individual basis. It is important to note that $\Gamma(N)$ does not reflect the impact of the N-th work on the individual. Instead, it summarizes the average performance of works created before and after the N-th

piece. To ensure a robust statistical representation, we adopted the definition $\Delta N = max(5, 0.1N_T)$ from the previous research, ensuring sufficient statistics when calculating $\Gamma(N)$. Additionally, to prevent overfitting, we introduced an L1 regularization term on the difference between *base* and each hot_1, hot_2, hot_3. 20 random initial values are prepared for the other parameters, adopting the one with the smallest squared error.

To examine the characteristics of fitting with the hot-streak model, we quantified the number of occurrences of hot streaks in a career. Consistent with previous research, Fig. S10a shows that one occurrence was most common, followed by two and zero occurrences, with three occurrences being the least common. Fig. S10b indicates that the distribution of hot streak lengths in years, remained constant across different career stages, averaging 4.1 years almost similar to previous studies. Lastly, we aggregated the start timing of hot streak in a career (Fig. S10c). Hot streaks were slightly more common in the early stages of the carriers, while the probability was about the same in the other periods. In previous studies, the probability of hot streak occurrence was equal for all carriers, which is a slight difference from the model reproduced here. These results indicate that we have reproduced the hot-streak model of a certain quality.

3.3 Dependency of the hot-streak model on window size of $\Gamma(N)$

In the context of successfully replicating the hot-streak model, we focused our attention on the window size that serves to smooth out the impact and found that the shape of $\Gamma(N)$ changes depending on the window size of the moving average ΔN. With the definition of $\Delta N = max(5, wsr * N_T)$, in which *wsr* represents window size ratio among each career, we then calculated $\Gamma(N)$ for career sequence data at four different *wsr* values. Looking at the two sequences as examples, the shape of $\Gamma(N)$ becomes smoother as *wsr* increases (Fig. S11).

To test the effect of *wsr*, we fitted a simple version of hot-streak model over the several $\Gamma(N)$ with four different *wsr*. The simple version assumes only one raise thus it has four parameters of *hot*, *base*, N_u and N_d. In Fig. S12, the impact elevation $Height$, calculated from $hot - base$, declines as *wsr* increases and the length of the hot streak, indicated by the difference between N_d and N_u, increases. This trend is confirmed by fitting the parameters for 500 researchers.

Notably, the distribution of the length of hot streaks *realtive L* peaks at the same length of *wsr*. This can be understood more clearly by examining the lower row of Figure S11. When career sequence data with a single outstanding impact are converted to $\Gamma(N)$, a shape resembling a plateau around the maximum impact emerges, whose length almost matches $wsr * N_T$, as intuitively obvious from the definition of the moving average. Fitting such $\Gamma(N)$ results in tracing a square-wave-pulse shape, suggesting a potential correlation between the relative length of the hot streak L and *wsr*. In such cases, the model detects a single significant success rather than consecutive successes. Given its current definition, the hot-streak model cannot prevent such false-positive detections. Therefore, there may be room for improvement in accurately detecting consecutive successes using the hot-streak model.

Indeed, there are cases where consecutive successes are detected, indicated by periods where the overall impact increases as shown in the upper row of Figure S11. The proposed hot-streak model undeniably contributes by capturing a common feature even among film directors and painters, which is the occurrence of consecutive hits. However, the main claims such as 1) 90% of researchers experience a hot streak, 2) hot streaks can occur at any time throughout a career, and 3) the average length of a hot streak is about four years, does not fully capture general trends applicable in all cases. This is because these are based on the distribution of fitting parameters when the window size for the moving average is arbitrarily assumed to be 10% of the career length.

3.4 The hot-streak model and our data-centric detection

To evaluate the correspondence between the replicated hot-streak model and our method, data-centric consecutive success detection, we examined the extent of alignment in the onset of hot streak periods. We normalized career timelines from 0 to 1 and defined the onset of a hot streak within a tolerance range of ±0.1 as a match. The comparison results are presented in a confusion matrix (Table. S1). Out of the consecutive success periods identified using our data-centric method, only 47% are also detected by the hot-streak model. This means our method overlooks roughly half of the periods caught by the model. Conversely, of the consecutive successes detected by the hot-streak model, only 19% are identified by the data-centric method. Given this, the hot-streak model tends to identify a

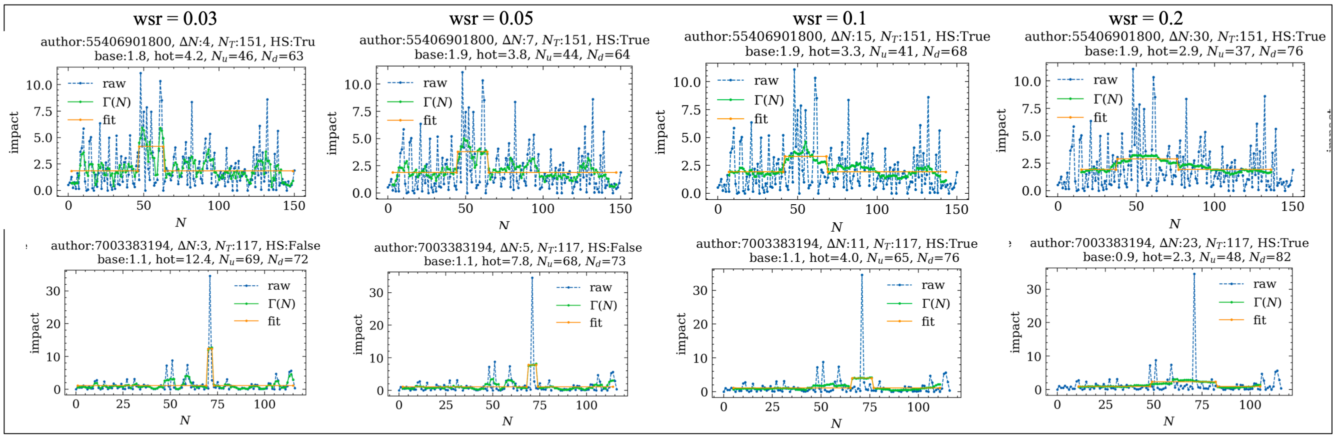

Figure S11. For two researchers, the moving average $\Gamma(N)$ (green) was calculated from the sequence data (blue), and a function assuming a single hot streak was fitted (orange). It can be observed that for the same data, as the window size increases, $\Gamma(N)$ becomes smoother and the fit goes lower and longer. The fitting was done by exploring all possible regions of four parameters under certain constraints, with the least squared error.

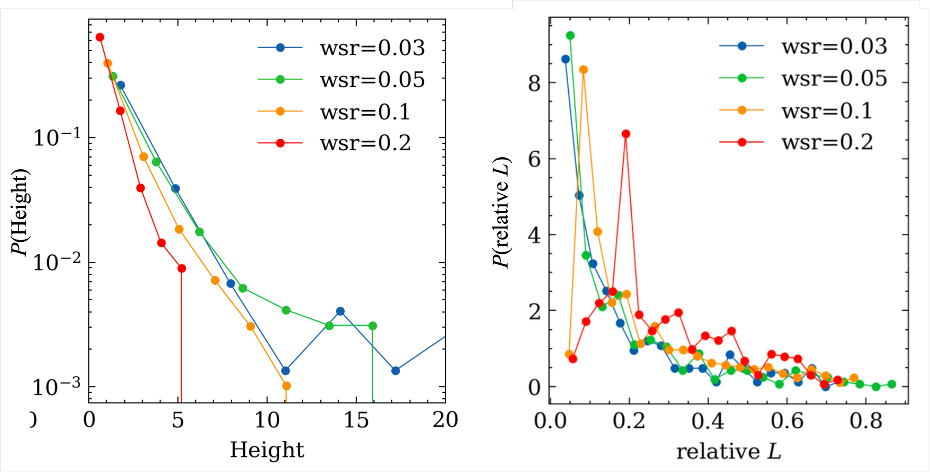

Figure S12. The distribution of parameters for the simple version of hot-streak model fitted to 500 researchers. As the window size increases, the height of the hot streak decreases, and its length increases. The height is defined as $hot - base$ and the length $realtiveL$ is the normalized length $(N_d - N_u)/N_T$.

significant portion of the periods as "success streaks" even when consecutive success of top-10% works is not occurring. Even in periods of consecutive success, it fails to recognize them about half the time (Fig. S13). In this study, we demonstrate the validity of our approach.

When employing the hot-streak model on sequences of raw impacts without applying a moving average, approximately 30% of researchers are found to experience at least one hot streak, while less than 10% undergo multiple hot streaks (Fig. S14a). This closely aligns with our model's ratio of X/N=3/5 and suggests that the 90% hot streaks identified in prior studies may be artifacts of high impacts being smoothed out by moving averages. Furthermore, while the average duration of hot streaks in models with a set window size is around four years, the variation is significantly greater in hot-streak models fitted to raw data (Fig. S14b). Comparing our method to the raw data fitting hot-streak model reveals that the latter does not fit well with non-consecutive successes. Comparing Tables S1 and S2, the number of hot streaks detected only by our data-centric method is fewer in the raw data fit. The count of hot streaks identified solely by the hot-streak model has increased. However, similar to the moving average, the distribution of the onset of hot streaks appears nearly flat(Figure S14c).

As limitations, due to computational constraints, the fitting was only performed on 500 individuals, and the method only considers up to a single hot streak, thus it is not a complete replication experiment. In the future, following previous studies, a method to fit up to three hot streaks will be applied to sufficient data of 100,000 individuals to observe more precise parameter distribution.

Our data-centric detection approach is simpler and directly engages with raw data, enabling a more accurate capture of real-world phenomena. The hot-streak model, on the other hand, works with career sequence data smoothed by moving averages. This model doesn't always successfully identify sequences of good work in a career, specifically the concentration of top-10% achievements, with only a 50% probability.

Table S1. The number of hot streaks detected by hot-streak model (n=100,000)

Hot-streak model	Data-centric detection: Positive	Data-centric detection: Negative
Positive	24,030	104,310
Negative	22,293	-

Table S2. The number of hot streaks detected by hot-streak model for raw data (n=100,000)

Hot-streak model for raw data	Data-centric detection: Positive	Data-centric detection: Negative
Positive	12,667	30,531
Negative	33,916	-

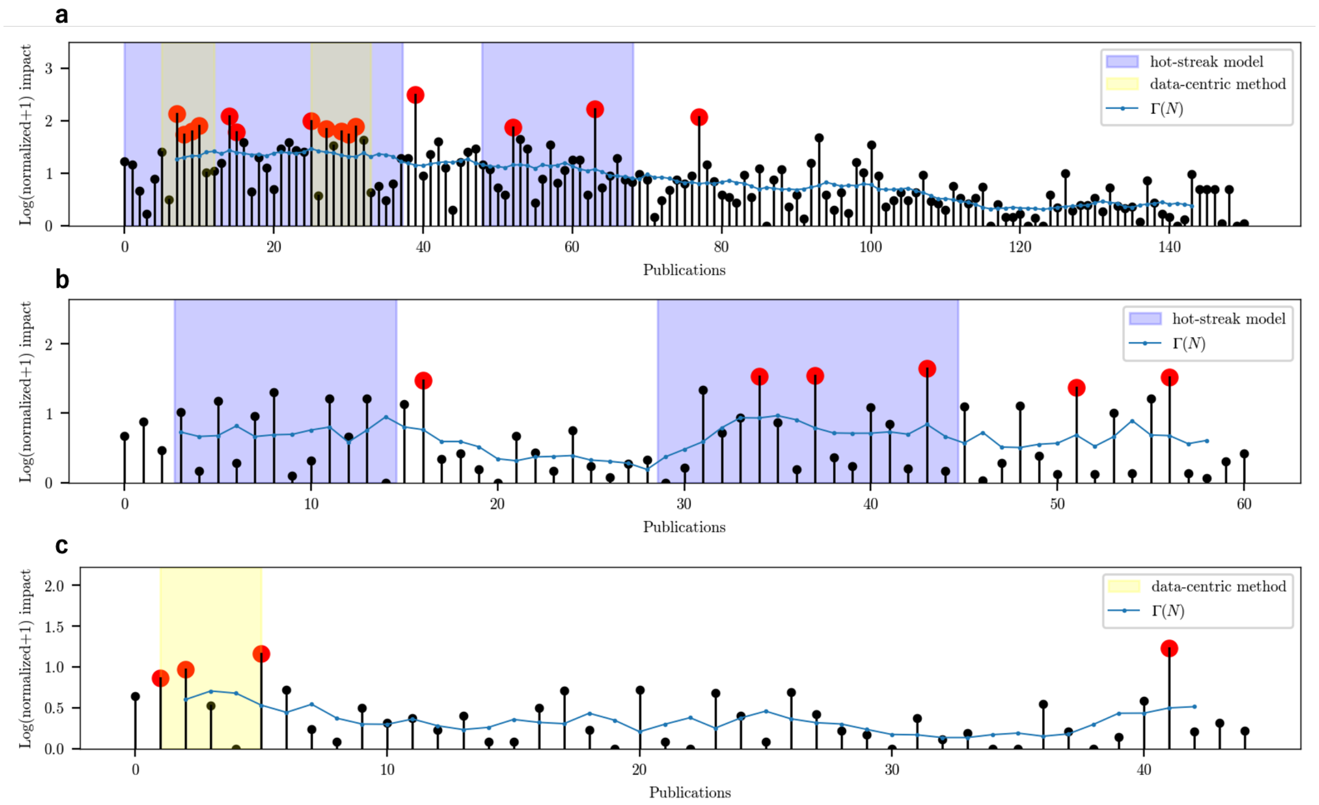

Figure S13. Differences in hot streak extraction trends between the hot-streak model and our data-centric approach. Red circles indicate the top-10% works in a career. **a**, When the timing of the hot streak was common. **b**, Cases in which the onset timing is not common and more than one hot streak was extracted by the hot-streak model. **c**, Cases where the onset timing was not common and one or more of them were extracted by the data-centric detection.

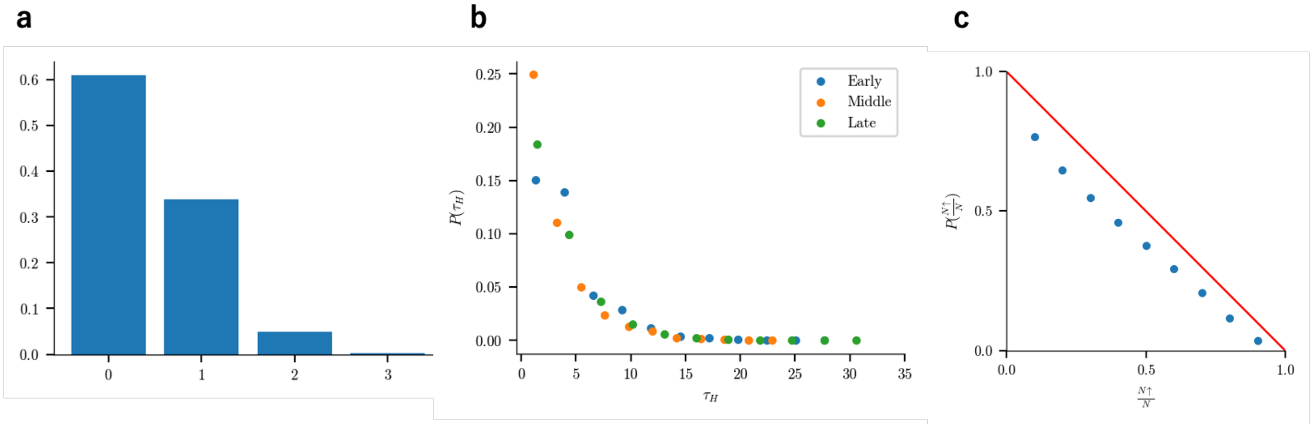

Figure S14. Characteristics of reproduced hot-streak model with raw-impact arrays. **a**, Histogram of the number of hot streaks in a career. **b**,The distributions of durations of hot streaks $P(\tau_H)$. **c**, The cumulative distributions the start of a hot streak.

4 Collaboration patterns

4.1 Team sizes

In sequences experiencing consecutive successes, 'Hot' sequences, team sizes tend to be larger, with a higher proportion of large projects involving more than 10 members (Fig. 2d,e). Histograms of team sizes for each sequence show similar proportions for teams up to 100 members, but 'Hot' sequences have a higher proportion of very large projects with over 100 members (Fig. S15). This indicates that large-scale projects often publish a cluster of papers at once, likely contributing to the top 10% high-impact papers in a career.

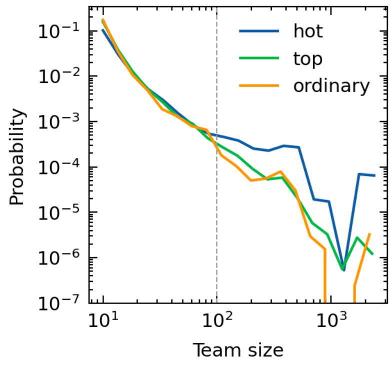

Figure S15. Histograms of team sizes for each sequence. The dashed line is on 100 of team size. 'Hot' sequences have greater proportion of big project.

4.2 Examining if early and late hot streaks come from the same work

We hypothesized that consecutive successes in early and late career stages may originate from the same teams. For instance, a researcher who had multiple hits as a student or postdoc in their early career might be linked to a mentor or principal investigator who appears as the last author during the same period. Therefore, these early-career mentors, often senior researchers later in their careers, could mirror the later career successes. To test this hypothesis, we sampled 20,000 top 10% papers in early hot streaks, defined as a relative career timing less than 0.2. Similarly, the same number of papers was also selected from the late hot streaks (greater than 0.8). If the overlap between these two sets of 20,000 papers is considerable than random sampling, the early and late consecutive successes are thought to have been brought about by mutual cooperation by early and senior researchers. The overlap was measured using the Jaccard index, which resulted in 0.002 between early and senior consecutive success. For comparison, the Jaccard coefficient was 0.1 for the pair of the sets of randomly extracted 20,000 papers regardless of career stage. This suggests that 'Hot' papers in early and late stages are more likely to be written by different teams, strongly supporting the notion that early and late career successes are qualitatively distinct. Notably, only 1.7% of individuals, 474 in total, experience consecutive successes in both the early and late stages of their career lifecycle.

4.3 Disruption index of hot works and works of who experience hot streak

Our findings indicate that scientists belonging to the early hot-streak group (experience hot streak up to 20% of the career timing) tend to experience less disruption in their scholarly careers compared to those in the middle (20-60% of the career timing), late (80% onward of the career timing) group. Furthermore, even in their hot streak papers, the early group displays low disruptiveness when compared to the other groups. These results suggest that early hot streaks are characterized by a limited level of topic diversity (see Figure S16). Scientists in this group tend to concentrate on developing their research and maintain a consistent style throughout their careers. Conversely, the late hot-streak group demonstrates relatively high levels of disruptiveness, despite also exhibiting low topic diversity (see Figure 2h). This can be attributed to their involvement in or leadership of large research teams and the presence of weak ties (see Figures 2d, f). These findings imply that they engage in research that incorporates diverse ideas and collaborations, thereby avoiding research with low levels of disruptiveness.

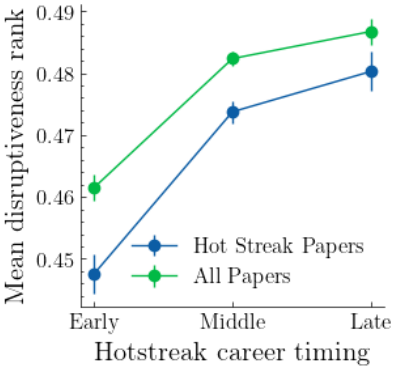

Figure S16. The blue line depicts the disruptiveness rank[13] (D) of hot streak papers during the early, middle, and late periods. The green line represents the D rank of all papers authored by scientists experiencing early, middle, and late hot streaks. The error bar for both series indicates a 95% confidence interval. The rank of D is determined within groupings that are classified according to year (in intervals of 5 years), cited count (divided into 5 percentiles), and the field (calculated in 1.1).

References

1. Baas, J., Schotten, M., Plume, A., Côté, G. & Karimi, R. Scopus as a curated, high-quality bibliometric data source for academic research in quantitative science studies. *Quant. Sci. Stud.* **1**, 377–386 (2020).
2. Miura, T., Asatani, K. & Sakata, I. Large-scale analysis of delayed recognition using sleeping beauty and the prince. *Appl. Netw. Sci.* **6**, 48 (2021).
3. Asatani, K., Oki, S., Momma, T. & Sakata, I. Quantifying progress in research topics across nations. *Sci. Reports* **13**, 4759 (2023).
4. Sinatra, R., Wang, D., Deville, P., Song, C. & Barabási, A.-L. Quantifying the evolution of individual scientific impact. *Science* **354**, aaf5239 (2016).
5. Radicchi, F., Fortunato, S. & Castellano, C. Universality of citation distributions: Toward an objective measure of scientific impact. *Proc. Natl. Acad. Sci.* **105**, 17268–17272 (2008).
6. Traag, V. A., Van Dooren, P. & Nesterov, Y. Narrow scope for resolution-limit-free community detection. *Phys. Rev. E* **84**, 016114 (2011).
7. Traag, V. A., Waltman, L. & Van Eck, N. J. From louvain to leiden: guaranteeing well-connected communities. *Sci. reports* **9**, 1–12 (2019).
8. Waltman, L. & Van Eck, N. J. A new methodology for constructing a publication-level classification system of science. *J. Am. Soc. for Inf. Sci. Technol.* **63**, 2378–2392 (2012).
9. Zeng, A. *et al.* Increasing trend of scientists to switch between topics. *Nat. communications* **10**, 3439 (2019).
10. Petersen, A. M., Pan, R. K., Pammolli, F. & Fortunato, S. Methods to account for citation inflation in research evaluation. *Res. Policy* **48**, 1855–1865 (2019).
11. Jones, B. F., Reedy, E. & Weinberg, B. A. Age and scientific genius. *The Wiley handbook genius* 422–450 (2014).
12. Liu, L. *et al.* Hot streaks in artistic, cultural, and scientific careers. *Nature* **559**, 396–399 (2018).
13. Wu, L., Wang, D. & Evans, J. A. Large teams develop and small teams disrupt science and technology. *Nature* **566**, 378–382 (2019).
